# Establishment of DNA Methylation Profile Associated with TCM Syndrome in Endometriosis

**DOI:** 10.1155/2022/4866360

**Published:** 2022-04-11

**Authors:** Xinyue Wang, Zhaofeng Zhang, Weiwei Zeng, Yuqing Zhong, Dandan Xie, Weiqiang Zhu, Fujia Chen, Jing Du, Tingting Zhang

**Affiliations:** ^1^Yueyang Hospital of Integrated Traditional Chinese and Western Medicine, Shanghai University of Traditional Chinese Medicine, Shanghai, China; ^2^NHC Key Lab of Reproduction Regulation (Shanghai Institute for Biomedical and Pharmaceutical Technologies), Fudan University, Shanghai, China; ^3^Shuguang Hospital, Shanghai University of Traditional Chinese Medicine, Shanghai, China

## Abstract

**Objectives:**

To screen the potential epigenetic biomarkers associated with endometriosis (EMS) and traditional Chinese medicine (TCM) syndrome EMS types.

**Methods:**

A cohort of 99 participants comprising 42 EMS patients with cold coagulation blood stasis (CCBS) syndrome, 35 EMS patients with Qi stagnation blood stasis (QSBS) syndrome, and 22 women of childbearing age without EMS were recruited. Reduced representation bisulfite sequencing (RRBS) was used to establish the differential DNA methylation profiles in human peripheral blood samples obtained from four non-EMS and four EMS patients with CCBS or QSBS syndrome, respectively. Differentially expressed genes (DEGs) were verified in 18 non-EMS, 38 CCBS-EMS, and 31 QSBS-EMS using pyrosequencing.

**Results:**

Methylation sites of 123942, 127229, and 115961 were found in peripheral blood DNA of non-EMS, CCBS-EMS, and QSBS-EMS patients, respectively. GO and KEGG analyses showed that the pathological process of EMS may be closely related to the nervous system development, cell junctions, GABA-gated chloride ion channel activity, nicotine addiction, Hippo signaling pathway, mRNA surveillance pathway, and Wnt signaling pathway. The methylation level at CpG site within HDAC6 gene in QSBS-EMS patients was significantly different from that in control women.

**Conclusions:**

The changes in DNA methylation in peripheral blood samples may be associated with EMS and TCM syndrome EMS types. The methylation level of HDAC6 gene may be used to distinguish QSBS-EMS patients from women without EMS.

## 1. Introduction

Endometriosis (EMS) is a common chronic gynecological disease occurring in women of reproductive age [1, 2]. It is characterized by the growth of endometrial, glandular, and stromal tissues outside the uterine cavity. Approximately 40–50% of patients experience infertility [3]. Present, several hypotheses from Western medicine propose explanations for EMS pathogenesis [4, 5], but none of these can explain the mechanism underlying EMS pathology. Based on EMS clinical manifestations, traditional Chinese medicine (TCM) considers it as “dysmenorrhea,” “Zheng Jia (blood stasis),” “irregular menstruation,” and “infertility” and believes that its underlying mechanism including its occurrence, development, and central link is “blood stasis; thus, the most basic pathological basis of EMS is “blood stasis.”

The treatment approaches for EMS in Western medicine are mainly surgery and drugs; however, the recurrence rate is high with these two measures [6]. The effects of treating EMS with TCM, such as Guizhi Fuling pills and Kuntai capsule, include a significant curative effect, low toxicity and side effects, long-term use of medication, and high pregnancy rates [7–9]. TCM treatments for EMS focus on promoting blood circulation and eliminating blood stasis [10]. EMS treatment strategies that “promote blood circulation and abrogate blood stasis” can exert good curative effects [11]. Our research team previously found that EMS patients often experience the symptoms of cold, which may be because the course of EMS is often long and blood activating drugs are used, making the patients susceptible to colds. This also causes prevention of blood stasis and production of heat, resulting in cold internally and heat externally [12].

Previous studies have shown that epigenetic pathways play important roles in the pathology of EMS [13–16] and the pharmacological mechanisms underlying TCM. Such pathways may thus be an important source of EMS-related biomarkers [17–19]. Genomics is very similar to the concept of constitution in TCM, and the essence of the syndrome may be revealed through the relationship between genomics and TCM syndrome. EMS, Qi stagnation blood stasis (QSBS), and cold coagulation blood stasis (CCBS) are common TCM syndrome types.

This study aimed to establish the differential profiles of DNA methylation in peripheral blood of patients with CCBS-type and QSBS-type EMS and to screen for potential biomarkers that may be useful to diagnose EMS and determine TCM syndrome. This will provide a reference for early clinical diagnosis of EMS and the development of personalized TCM treatment approaches, providing new methods to study the pathological mechanism of EMS.

## 2. Materials and Methods

### 2.1. Participants and Samples

With approval from the Ethics Committee of Yueyang Integrated Traditional Chinese and Western Medicine Hospital Affiliated to Shanghai University of Traditional Chinese Medicine (nos. 2018-082, 2019-041) and from the patients using the informed patient consent, 42 CCBS-type and 35 QSBS-type EMS patients who visited the gynecology department of the hospital from September 2018 to June 2020 were selected. These 77 participants were diagnosed with EMS and were aged 20–45 years. With the approval of the Medical Ethics Committee of Shanghai Institute of Family Planning Science No. PJ2018-0818, 22 healthy, nonpregnant women of childbearing age were selected as controls. Patients' diagnoses were according to the 2019 guidelines for the diagnosis and treatment of endometriosis with integrated traditional Chinese and Western medicine [20] and the 2015 guidelines for the diagnosis and treatment of endometriosis [21].

### 2.2. Reduced Representation Bisulfite Sequencing (RRBS)

Total genomic DNA was extracted from blood samples using the EasyPure® Genomic DNA Kit according to the manufacturer's instructions. Genome-wide DNA methylation profiles from four non-EMS, four CCBS-EMS, and four QSBS-EMS were examined using RRBS. Briefly, library construction was completed using the TruSeq® DNA LT Sample Prep Kit v2 (Illumina). Extracted DNA of 500 ng was digested using MspI. DNA fragments were processed by 3' and repair, A' tailing, and followed by ligation with barcoded methylated TruSeq LT adapters (Illumina). After bisulfite disposal using the Qiagen EpiTect kit, PCR amplification was performed. Multiplexed samples were pooled into a DNA library, and 100 bp paired-end sequencing was performed on HiSeq-2500 (Illumina). Quality control analysis of sequencing reads was conducted using FastQC software 7 (https://www.bioinformatics.babraham.ac.uk/projects/fastqc/available from) [22].

### 2.3. Construction of Functionally Enriched Pathways and PPI Network

Gene Ontology (GO) analysis was conducted using Gene Ontology Consortium (https://www.geneontology.org/), and the analysis of pathway enrichment in EMS was performed in accordance with the Kyoto Encyclopedia of Genes and Genomes (KEGG) pathway database. Search Tool for the Retrieval of Interacting Genes (STRING) was applied to analyze protein-protein interactions among genomes with a confidence coefficient threshold of >0.7. PPI network was visualized using Cytoscape software (ver. 3.3.0, https://www.cytoscape.org/) and molecular complex detection (MCODE). MCODE score was >4. Functional enrichment analysis of the genomes from single modules was conducted using DAVID when *P* values were <0.05 (https://david.abcc.ncifcrf.gov/home.jsp) [23].

### 2.4. Pyrosequencing to Quantify Differentially Expressed Genes (DEGs)

Sizeable, significant (*P* value <0.05 and |log2FC|>0.8, FC, fold change) DMRs were tested using pyrosequencing assay. Extracted DNA methylation was assessed using the Qiagen EpiTect Bisulfite kit (Qiagen, 59104). PCR amplification (ABI 9700 PCR System, Applied Biosystems) for 40 cycles was performed. Pyrosequencing was performed using PyroMark Q96/48 ID (Qiagen), and the final datasets were calculated using PyroQ CpG software (Biotage) according to the manufacturer's protocols [24].

### 2.5. Statistical Analysis

SPSS software (version 23.0) was used to analyze the data. Measured data were expressed as means ± standard deviation (*x* ± *s*). One-way ANOVA was used for comparison among multiple groups and nonparametric tests were used for data that did not conform to normal distribution and/or homogeneity of variance. *P* value was bilateral, and *P* < 0.05 was considered significant.

## 3. Results

### 3.1. Comparison of Subjects' Age in Each Group

According to TCM syndrome differentiation criteria, 42 and 35 cases of the EMS patients were designated to the EMS CCBS group and QSBS-EMS group, respectively. No significant difference in average subject age was found between groups (*P* < 0.05) (Table 1).

### 3.2. Identification of Aberrant DMGs among CCBS and QSBS-EMS Patients and Healthy Individuals

**Figure 1:**
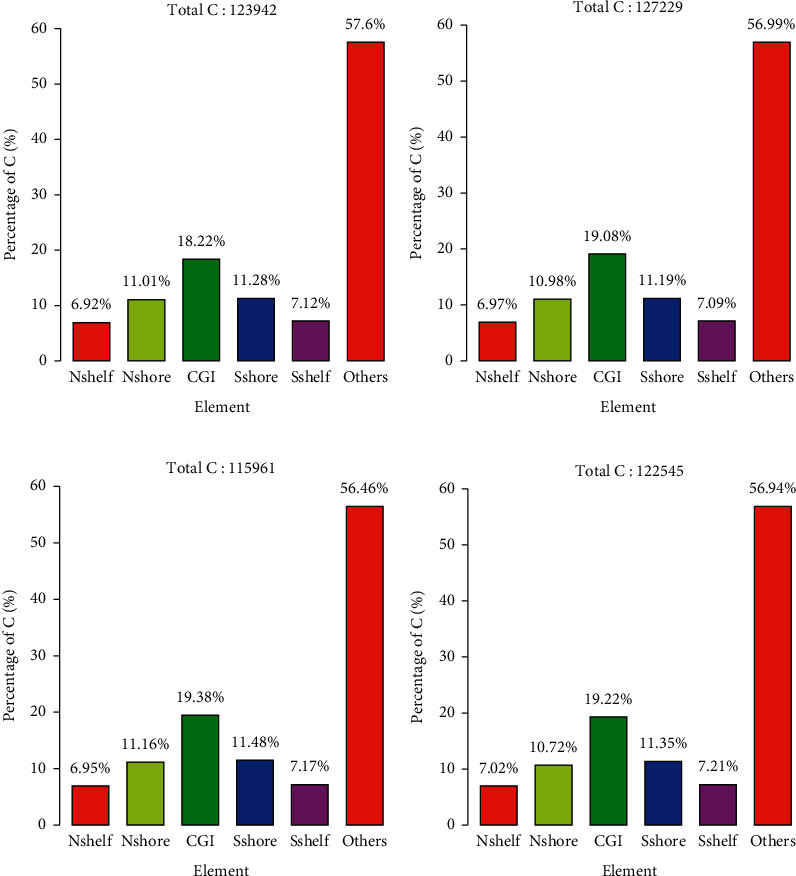
Distribution of differential loci in gene functional elements. (a) Non-EMS vs. EMS. (b) Non-EMS vs. CCBS-EMS. (c) Non-EMS vs. QSBS-EMS. (d) CCBS-EMS vs. QSBS-EMS. Nshelf, 2–4 kb upstream of CpG island; Nshore, 0–2 kb upstream of CpG island; CGI, CpG island, Sshore, 0–2 kb downstream of CpG island; Sshelf, 2–4 kb downstream of CpG island.

Using RRBS genome-wide methylation high-throughput sequencing technology, compared to women without EMS, we identified 123942, 127229, and 115961 differentially methylated loci in the peripheral blood DNA of women with EMS, CCBS-EMS, and QSBS-EMS, respectively. There were also 122545 differentially methylated loci between CCBS-EMS and QSBS-EMS patients (Figure 1).

### 3.3. Functional and Pathway Enrichment Analyses

**Figure 2:**
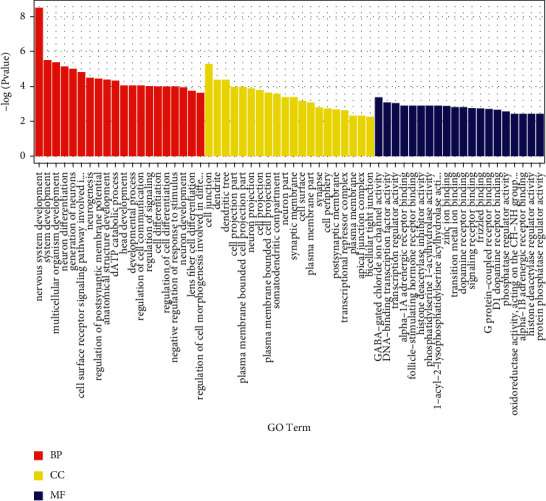
Results of go enrichment analysis of differentially expressed genes among CCBS, QSBS-EMS, and non-EMS.

**Figure 3:**
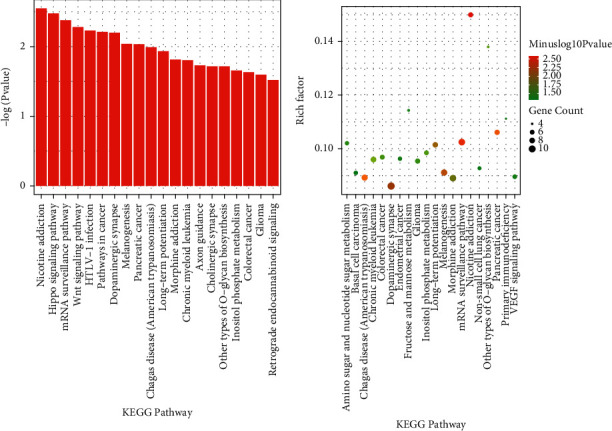
KEGG analysis of differentially expressed genes among CCBS, QSBS-EMS, and non-EMS.

GO functional analysis showed that DEGs were useful in nervous system development, cell junctions, and GABA-gated chloride ion channel activity (Figure 2). KEGG enrichment analysis indicated that DEGs were related to nicotine addiction, Hippo signaling, mRNA surveillance, and Wnt signaling (Figure 3).

### 3.4. Pyrophosphate Verification Results for the Methylation Level of GPR50 and HDAC6 Genes

**Figure 4:**
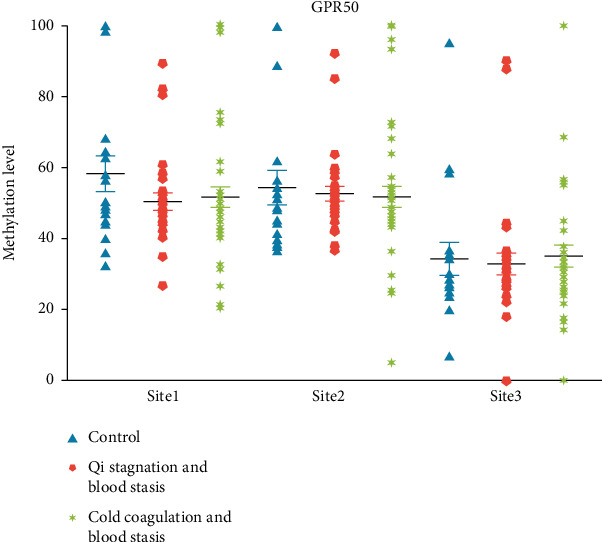
Verification results of GPR50 gene methylation level of subjects in each group. Control group, healthy women of childbearing age without EMS (*n* = 18); QSBS, patients with EMS of QSBS type (*n* = 31); cold coagulation blood stasis, EMS patients of CCBS type (*n* = 38).

After comparing the differentially methylated genes with GO and KEGG analyses results for non-EMS, CCBS-EMS, and QSBS-EMS groups, we verified and compared the DNA methylation levels of three different sites on the CGI of GPR50 gene in peripheral blood of non-EMS, QSBS-EMS, and EMS-CCBS groups. The results showed no significant difference (Table 2 and Figure 4).

**Figure 5:**
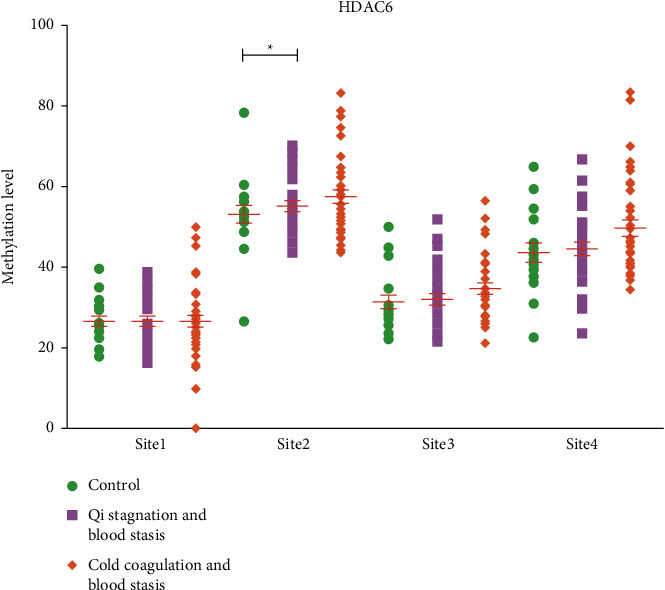
Verification results of HDAC6 gene methylation level of subjects in each group. Control group, healthy women of childbearing age without EMS (*n* = 18); QSBS, patients with EMS of QSBS type (*n* = 31); cold coagulation blood stasis, EMS patients of CCBS type (*n* = 38). ^*∗*^*P* < 0.05, compared with the control group.

We also verified and compared the DNA methylation levels of four different sites on CGI of HDAC6 gene in peripheral blood of non-EMS, QSBS-EMS, and CCBS-EMS groups. The results revealed a significant difference in site 2 between QSBS-EMS and non-EMS groups, indicating that methylation was lower in the QSBS group than in the non-EMS group. No significant differences were found among the other sites (Table 3 and Figure 5).

## 4. Discussion

In this study, we comprehensively analyzed the differences in the levels of peripheral blood DNA methylation between non-EMS, CCBS-EMS, and QSBS-EMS groups. Using RRBS genome-wide methylation sequencing, compared to the non-EMS group, we screened 123942, 127229, and 115961 differential loci in the peripheral blood DNA of EMS, CCBS-EMS, and QSBS-EMS patients, respectively, of which about 24% were distributed in CGI or LTR regions. There were also 122545 differentially methylated loci between CCBS-EMS and QSBS-EMS, and 25% of these loci were distributed in CGI or LTR region. These results suggest that there may be many differentially expressed genes among these groups.

GO enrichment analysis identified the functions of the differentially expressed genes, which may be related to connections between cells, especially neurons, and cell metabolic functions. KEGG analysis suggested that methylation differences may cause primary immune deficiency and O-glycan biosynthesis. The common pathways in CCBS-EMS and QSBS-EMS included melanin production and dorsal ventral axis formation pathways, and the different pathways included fatty acid synthesis, pancreatic secretory activity, and hedgehog signaling pathways.

Yes-associated protein (YAP) is a central downstream effector of the Hippo signaling pathway, which is involved in cell proliferation, differentiation, and apoptosis. The expression of YAP is upregulated in the ectopic and eutopic endometria of EMS patients [25]. The decrease in autophagy level is related to the increase in YAP expression [19]. Secretory curl associated protein 2 (SFRP2) and catenin *β*1 (CTNN *β*1) regulate the growth of EMS lesions and indicate the boundary of lesions, which are the typical stimulants of the Wnt/*β*-catenin signaling pathway [26]. Studies have shown that MRP4 gene in mouse uterine tissue can inhibit the expression of Wnt/*β*-catenin target genes in EMS [27], and forkhead box protein P1 (FOXP1) can promote the fibrosis of EMS by upregulating Wnt signal activity [28].

Differentially methylated genes in peripheral blood of EMS patients are likely to be related to nerve function and the activities of Hippo and Wnt signaling pathways, indicating that these changes may be related to the pathological mechanism of EMS. In particular, the pathology of CCBS-EMS and EMS may be closely related to immune and nervous systems functions. We speculate that the complex interactions between the immune system, nerves, blood vessels, local inflammation, sensory nerve hypersensitivity, and hyperalgesia are important contributors of dysmenorrhea in CCBS-EMS and EMS. The main underlying cause of CCBS syndrome in TCM is improper diet and lifestyle, such as exposure to cold and rain, excessive eating, or excessive consumption of raw and cold substances, causing injury to the internal organ due to cold. Cold stagnation can easily block Qi, blood, and body fluids. Blood stasis can occur in the uterus and Chong-Ren can be obstructed. Cold dominates pain and cold exposure hurts the body, which most likely damages Yang Qi of the body. If Qi and blood channels lose their warmth, their functions will be hindered, causing blood to coagulate and stagnate, and this is usually painful. Pain due to cold generally has two characteristics: one is that the patient has obvious signs of cold exposure and the other is that the degree of pain is reduced in warm temperature and aggravated by cold. Among the six evils, cold has the closest relationship with pain. Our experimental results may provide evidence supporting the TCM theory.

After verification, we found that there was a significant difference in the methylation level of site 2 within HDAC6 gene between the QSBS-EMS group and non-EMS group. The HDAC gene family encodes histone deacetylases whose main function is to regulate chromosome structure modification and gene expression. HDAC6 is a member of class II HDAC family and a major cytoplasmic enzyme. It can participate in a variety of biological processes by regulating lysine acetylation and related cellular pathways, and it plays important roles in regulating gene expression, cell cycle processes, autophagy, and apoptosis. Abnormal expression of HDAC6 contributes to the pathological mechanisms of a variety of malignant tumors [29, 30].

Studies have shown that tamoxifen-resistant T47D (T47D-TAR) cells are sensitive to HDAC6 inhibition by its effect on the Hippo pathway and inhibiting HDAC6 by suppressing YAP expression is considered a potential new strategy for treating tamoxifen-resistant breast cancer. Abnormal upregulation of HDAC6 expression can also decrease *β*-catenin signaling, thereby inhibiting cell proliferation and migration [31]. In addition, HDAC6 upregulation can inhibit the mesenchymal transformation of hepatocellular carcinoma (HCC) epithelial cells by increasing the level of E-cadherin protein and reducing the levels of N-cadherin, vimentin, and MMP-9 proteins [32]. Notably, HDAC6 activity also correlates with emotional state and immune function. HDAC6 knockout can effectively reverse the phenotype for depression in mice. HDAC6 gene-deficient mice show less anxiety, more hyperactivity, and less depression, and HDAC6 specific inhibitors have antidepressant-like effects in mice [33, 34]. Other studies have confirmed that HDAC6 is involved in cell migration, immune synapse formation, and intercellular interactions [35], and HDAC6 overexpression leads to immune synapse disorders, suggesting that it may be related to the development of autoimmune diseases [36, 37].

EMS-QSBS is mainly caused by depression, anger, and anxiety in TCM. TCM believes that the liver plays an important role in regulating emotions. Abnormal mood leads to the stagnation of liver Qi and liver failure to maintain normal Qi flow. Abnormal blood circulation often occurs when Qi is not flowing smoothly and its stagnation causes blood stasis, dysmenorrhea, late menstruation, and accumulation of ruffian mass. We speculate that QSBS-EMS patients have a more common and stronger tendency of having depression and/or anxiety than patients with other syndrome types. Therefore, we further believe that abnormal HDAC6 gene expression caused by an abnormal HDAC6 methylation level may be one of the pathological causes of immune system disorders and emotional depression in QSBS-EMS patients. In the GO and KEGG analyses, QSBS-EMS type was not found to correlate with immune function. We think it is necessary to increase the sample size and the number of EMS patients with other TCM syndrome types in future research to further explore the occurrence and the mechanism underlying the development of EMS in association with other TCM syndrome types.

This study has two limitations. First, the mechanisms relating signal pathways to pathological processes in different types of EMS were not experimentally confirmed. Second, the sample size was relatively small. Larger sample sizes are needed for further experimental validation of DEGs and their expression.

## 5. Conclusion

This study systematically elucidated the unique observed changes in peripheral blood DNA methylation levels in CCBS-EMS and QSBS-EMS patients and confirmed the existence of many differences in peripheral blood DNA methylation levels between CCBS-EMS and QSBS-EMS patients.

This study also found that the pathological mechanism of CCBS and QSBS-type EMS may be related to disorders in the nervous system development and immune system function, but there may be differences in pathological characteristics including metabolic function and emotional influence, and HDAC6 may be a potential molecular marker of QSBS-type EMS.

## Figures and Tables

**Table 1:** Average age of subjects in each group.

Group	Numbers	Age
Non-EMS group	22	28.13 ± 3.81
CCBS-EMS group	42	34.95 ± 5.42
QSBS-EMS group	35	33.80 ± 4.27
*P*	>0.05	

**Table 2:** Average methylation level of three loci on CGI of GPR50 gene in each group (%).

Group	Numbers	Site 1	Site 2	Site 3
Non-EMS group	18	53.49 ± 15.36	52.08 ± 12.75	33.73 ± 11.30
CCBS-EMS group	31	48.45 ± 17.65	49.63 ± 16.72	32.28 ± 16.63
QSBS-EMS group	38	55.83 ± 17.86	55.52 ± 17.70	36.07 ± 22.24
*P*		>0.05	>0.05	>0.05

**Table 3:** Average methylation level of 4 sites on CGI of HDAC6 gene in each group (%).

Group	Numbers	Site 1	Site 2	Site 3	Site 4
Non-EMS group	18	28.49 ± 7.23	60.11 ± 10.21	35.42 ± 9.96	50.27 ± 11.83
CCBS-EMS group	31	26.57 ± 9.38	53.40 ± 8.78^*∗*^	30.56 ± 7.91	44.93 ± 11.75
QSBS-EMS group	38	25.85 ± 6.99	55.81 ± 8.37	34.29 ± 7.55	46.51 ± 9.94
*P*		>0.05	<0.05	>0.05	>0.05

^*∗*^ *P* < 0.05, compared with the control group.

## Data Availability

The data used to support the findings of this study are available from the corresponding author upon request.
